# Outcome and quality of life in patients with postoperative delirium during an ICU stay following major surgery

**DOI:** 10.1186/cc13084

**Published:** 2013-10-29

**Authors:** Fernando J Abelha, Clara Luís, Dalila Veiga, Daniela Parente, Vera Fernandes, Patrícia Santos, Miguela Botelho, Alice Santos, Cristina Santos

**Affiliations:** 1 Department of Anesthesiology, Centro Hospitalar de São João, Faculty of Medicine, University of Porto, Alameda do Professor Hernani Monteiro, 4202-451, Porto, Portugal; 2 Health Information and Decision Sciences Department, Faculty of Medicine, University of Porto, Alameda do Professor Hernani Monteiro, 4202-451, Porto, Portugal; 3 Anesthesiology and Perioperative Care Unit, Surgical Department, Faculty of Medicine, University of Porto, Alameda do Professor Hernani Monteiro, 4202-451, Porto, Portugal

## Abstract

**Introduction:**

Delirium is an acute disturbance of consciousness and cognition that has been shown to be associated with poor outcomes, including increased mortality. We aimed to evaluate outcome after postoperative delirium in a cohort of surgical intensive care unit (SICU) patients.

**Methods:**

This prospective study was conducted over a 10-month period in a SICU. Postoperative delirium was diagnosed in accordance with the Intensive Care Delirium Screening Checklist (ICDSC). The primary outcome was mortality at 6-month follow-up. Hospital mortality and becoming dependent were considered as secondary outcomes, on the basis of the evaluation of the patient’s ability to undertake both personal and instrumental activities of daily living (ADL) before surgery and 6 months after discharge from the SICU. For each dichotomous outcome - hospital mortality, mortality at 6-month follow-up, and becoming dependent - a separate multiple logistic regression analysis was performed, which included delirium as an independent variable. Another outcome analyzed was changes in health-related quality of life, as determined using short-form 36 (SF-36), which was administered before and 6 months after discharge from the SICU. Additionally, for each SF-36 domain, a separate multiple linear regression model was used for each SF-36 domain, with changes in the SF-36 domain as a dependent variable and delirium as an independent variable.

**Results:**

Of 775 SICU-admitted adults, 562 were enrolled in the study, of which 89 (16%) experienced postoperative delirium. Delirium was an independent risk factor for mortality at the 6-month follow-up (OR = 2.562, *P* <0.001) and also for hospital mortality (OR = 2.673, *P* <0.001). Delirium was also an independent risk factor for becoming dependent for personal ADL (P-ADL) after SICU discharge (OR = 2.188, *P* <0.046). Moreover, patients who experienced postoperative delirium showed a greater decline in SF-36 domains after discharge, particularly in physical function, vitality, and social function, as compared to patients without postoperative delirium.

**Conclusions:**

Postoperative delirium was an independent risk factor for 6-month follow-up mortality, hospital mortality, and becoming independent in P-ADL after SICU discharge. It was also significantly associated with a worsening in the quality of life after surgery.

## Introduction

Delirium, which is also referred to as an ‘acute confusional state’, is a transient, global cognitive disorder. After surgery, delirium typically develops after one to four days postoperatively [1] and affects approximately 10 to 70% of patients older than 65 years [2]. Postoperative delirium is associated with high morbidity and mortality rates, and prolonged stays in the hospital, the intensive care unit (ICU), and the post-anesthesia care unit [3-5]. In the ICU, postoperative delirium often goes unnoticed, as it is regarded as commonplace after surgery, or an expected and inconsequential outcome of mechanical ventilation and other necessary life-saving treatments [6].

Given the high prevalence of delirium among critically ill patients, and its associated negative clinical outcomes, current practice guidelines recommend that ICU patients be routinely screened for delirium by using a validated screening tool such as the Confusion Assessment Method for the ICU (CAM-ICU) or the Intensive Care Delirium Screening Checklist (ICDSC) [7]. Delirium has been associated with cognitive decline and older age [8-11]. Lipowski [12] stated that delirium may increase the risk of mental deterioration, and there is some evidence that patients who experience postoperative delirium represent a subgroup at risk of prolonged and even permanent cognitive disorders that may negatively affect their quality of life [13]. Although reports of alterations in cognitive function after delirium are available in the literature, to our knowledge, there are no published studies examining the impact of postoperative delirium on health-related quality of life (HRQL).

The aim of our study was to evaluate the association of postoperative delirium with different outcomes and changes in HRQL. The primary outcome was mortality at six-month follow-up. As secondary outcomes, we considered hospital mortality, changes in HRQL, and becoming dependent with regard to the ability to undertake both personal and instrumental activities of daily living (ADL) before surgery and six months after discharge from a surgical intensive care unit (SICU).

## Materials and methods

### Patient recruitment and parameters

The institutional review board and the ethics committee of the Hospital of São João, Porto, Portugal, approved the study; informed consent was obtained preoperatively from each patient. This prospective cohort study was performed in the post-anesthesia care unit (PACU) of the Hospital of São João. Within the PACU, there is a surgical intensive care unit (SICU) with five beds, where critically ill patients are admitted, closely monitored, and treated.

The subjects were recruited over a 10-month period between November 2008 and August 2009. All of the scheduled postoperative patients considered eligible for the study were Portuguese-speaking adults who were admitted to the SICU for major noncardiac and nonneurological surgeries requiring anesthesia and a postoperative hospital stay of more than 48 hours. Patients who underwent emergency surgery or did not provide informed consent were excluded from the study, as were those with a history of central nervous system disease, Parkinson’s disease, neurological or cardiac surgery, delirium or antipsychotic medication, or drug abuse (including alcohol abuse).

The following variables were recorded on admission to the SICU: age, sex, body mass index (BMI), and preadmission comorbidities, specifically ischemic heart disease, congestive heart failure, cerebrovascular disease, hypertension, renal insufficiency, diabetes, and hyperlipidemia. The anesthesia data collected for each patient consisted of information on American Society of Anesthesiologists Physical Status (ASA-PS), duration and type of anesthesia, amount of crystalloids administered during surgery, and frequency of use of colloids and blood products. SICU data, length of hospital stay (LOS), and mortality were also recorded for all the patients.

The Acute Physiology and Chronic Health Evaluation (APACHE) II score [14] and the Simplified Acute Physiology Score II (SAPS II) [15] were calculated using standard methods. The Revised Cardiac Risk Index (RCRI) was calculated using the classification system reported by Lee *et al.*[16]. Troponin I levels were also recorded on admission and for each day in the SICU. The mortality data were derived from the records of registered SICU mortality, hospital mortality, and mortality at six months after SICU discharge.

### Delirium evaluation

Sedation levels were evaluated using the Richmond Agitation and Sedation Scale (RASS) [17]. The ICDSC was only administered if the level of sedation as assessed by the RASS was between −3 and +4. Each patient admitted to the SICU and included in the study was evaluated prospectively by research staff physicians and one of the bedside nurses to establish a diagnosis of delirium, using the ICDSC [18]. The ICDSC consists of eight items based on the Diagnostic and Statistical Manual of Mental Disorders (DSM) criteria and features of delirium, including inattention, disorientation, hallucination (delusion psychosis), psychomotor agitation or retardation, inappropriate speech or mood, sleep/wake cycle disturbances, and symptom fluctuation.

Scores were assigned to each ICU patient by a nurse during every shift. The patients were assessed on the basis of the ICDSC at least once every 8 h for the entire duration of their SICU stay. All patients that scored 4 or higher in the ICDSC at least once were considered to have delirium.

### Medical outcomes study short form 36 (SF-36)

HRQL was assessed using the Medical Outcomes Study Short Form 36 (SF-36) [19], which was completed directly by the patients before surgery and six months after discharge from the SICU.

The SF-36 consists of eight sections or domains, which are the weighted sums of the questions in their section. The eight domains are vitality, physical functioning, bodily pain, general health perceptions, physical role functioning, emotional role functioning, social role functioning, and mental health. Patient records were checked against hospital records after six months to determine whether a patient was still alive before being considered for inclusion in the long-term postoperative follow-up aspect of the study. A formal letter, a validated Portuguese SF-36 self-report form, and a return envelope were sent to all known survivors [20].

### Functional capacity

An evaluation of functional capacity, based on the ability of the patient to undertake personal and instrumental ADL, was performed before surgery. For all patients, this same evaluation was repeated six months after SICU discharge. A questionnaire that evaluates the functional independence of the individual with respect to performance of personal ADL (P-ADL) and instrumental ADL (I-ADL) was used based on Katz’s Index of Independence in ADL [21] and the Lawton I-ADL [22] scale. The Lawton I-ADL scale is an easily administrable assessment tool that provides self-reported information about the functional skills necessary for living in the community. Deficits in this scale were recorded and a summary score ranging from 0 (dependent) to 7 (independent) was obtained. The Katz ADL scale assesses basic personal activities of daily living, and ranks adequacy of performance in six functions. Dependency while performing each personal activity was evaluated and a summary score ranging from 0 (independence in all activities) to 6 (dependency in all activities) was obtained. The P-ADL functions were bathing, dressing, going to the toilet, transferring from the bed to a chair, continence, and eating. We considered the following activities as I-ADL functions: using the telephone, shopping, housekeeping, food preparation, using public transport, handling finances, and effectively taking responsibility for their own medications.

The patients’ responses were categorized into two groups: able or unable to perform each activity or group of activities. Patients were considered to be dependent if they were dependent with regard to any I-ADL or P-ADL activity. We further evaluated the patients who became dependent in I-ADL and P-ADL after the SICU stay.

### Outcomes

The primary outcome was mortality at six-month follow-up. We considered hospital mortality as a secondary outcome. We also considered changes in each domain of the HRQL as outcomes. The changes in HRQL were computed on the basis of the differences between the findings in each of the eight domains of the SF-36 before surgery, and six months after discharge from the SICU. Dependency with regard to P-ADL and I-ADL were also considered as outcomes. We considered that patients became P-ADL-dependent if they were not dependent before surgery but were dependent at the time of the six-month evaluation. Similarly, we considered that patients became dependent for I-ADL if they were not dependent before surgery but were dependent at the time of the six-month evaluation. All data relating to functional status and quality of life before surgery were obtained directly from the patients before surgery.

### Statistical analyses

Before admission, patient characteristics that were likely to affect outcomes were recorded. These included age, gender, ASA-PS, BMI, duration and type of anesthesia, emergency surgery, temperature and troponin I at SICU admission, hypertension, hyperlipidemia, chronic obstructive pulmonary disease (COPD), high-risk surgery, ischemic or congestive heart disease, cerebrovascular disease, renal insufficiency, insulin therapy for diabetes, total RCRI, and administration of crystalloids, colloids, erythrocytes, fresh frozen plasma, and platelets. Preadmission variables of patients who exhibited postoperative delirium were compared with those of patients who did not, using the Mann-Whitney *U* test and chi-square test.

For each dichotomous outcome - hospital mortality, mortality at the six-month follow-up, and dependency (I-ADL and P-ADL) - a separate multiple logistic regression model was used considering each outcome as a dependent variable and delirium as an independent variable, among other preadmission variables. Some independent variables, such as age and APACHE score, were included in the models as *a priori* knowledge. Variables correlated with outcomes at *P* <0.2 (chi-square test or Mann-Whitney *U* test) were also included in the respective model as independent variables, together with delirium and the other variables selected from *a priori* knowledge, in accordance with the methods described by Greenland [23].

For each SF-36 domain, a separate multiple linear regression analysis was performed with changes in the SF-36 domain considered as dependent variables and delirium as an independent variable, together with other independent variables predetermined to be included in the models as *a priori* knowledge. We performed no model reduction in the multivariate analyses, in accordance with methods described by Greenland [23,24].

Linear regression models were checked using Q plots to assess the normality of residuals from the linear models, and a *t* test was used to determine if mean residuals were equal to zero. Also the homoscedasticity was verified by plotting residuals against the fitted values. Analysis of multicolinearity between independent variables was also performed using a variance inflation factor (VIF), to avoid the inclusion of highly correlated independent variables. A significance level of 5% was predetermined. The data were analyzed using SPSS version 19.0 for Windows (SPSS Inc., Chicago, IL, USA).

## Results

**Figure 1:**
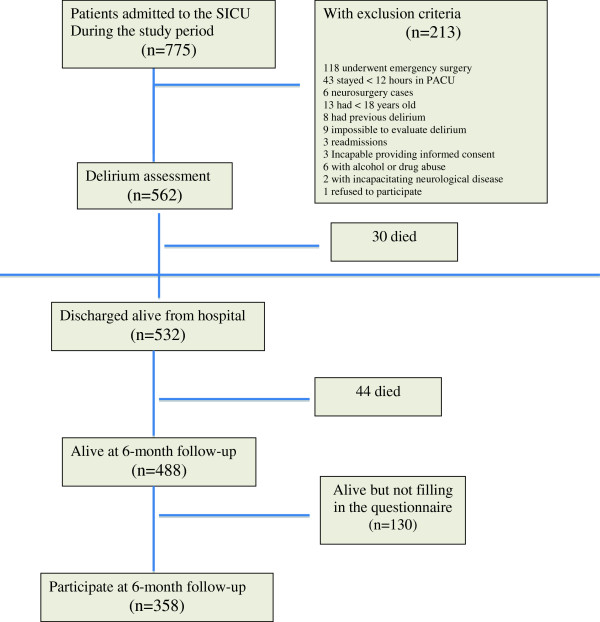
Flow diagram.

Of 775 adults admitted to the SICU during the study period, 562 were included in the study (Figure 1), of which 89 (16%) experienced delirium after SICU admission. The characteristics of the patients who did and did not exhibit delirium are summarized in Table 1. Mortality was higher in patients with delirium than in those without delirium in the SICU, in the hospital, and at the six-month follow-up (Table 1). Mortality was 5% (30 patients) at hospitalization, and 13% (74 patients) at the six-month follow-up.

**Table 1:** Preadmission patient characteristics and outcomes

	**All**	**No delirium**	**Delirium**	
	**(n = 562)**	**(n = 473) 84%**	**(n = 89) 16%**	***P***
Admission and preadmission patient characteristics:				
Age in years, median (IQR)	66 (54-74)	64 (53-73)	73 (61-81)	<0.001^a^
Age group, n (%)				<0.001^b^
65 years	284 (51)	223 (47)	61 (69)	
<65 years	278 (49)	250 (53)	28 (32)	
Gender, n (%)				0.346^b^
Male	354 (63)	294 (62)	60 (67)	
Female	208 (37)	179 (38)	29 (33)	
ASA physical status, n (%)				<0.001^b^
I/II	189 (34)	175 (37)	14 (16)	
III/IV	373 (66)	298 (63)	75 (84)	
Body mass index in kg/m^2^, median (IQR)	25 (23-28)	25 (23-28)	26 (23-29)	0.231^a^
Duration of anesthesia (min.), median (IQR)	240 (180-330)	240 (180-330)	240 (180-330)	0.343^a^
Type of anesthesia, n (%)				0.903^a^
General/combined general locoregional	476 (85)	401 (85)	75 (84)	
Locoregional	86 (15)	72 (15)	14 (16)	
Temperature at SICU admission, median (IQR)	35.0 (33.9-35.8)	35.0 (33.9-35.8)	35.0 (34.0-35.6)	0.905^a^
Troponin I at SICU admission, median (IQR)	0.01(0.01-0.02)	0.01(0.01-0.01)	0.01(0.01-0.02)	0.001^a^
Hypertension, n (%)	341 (61)	275 (58)	66 (74)	0.005^b^
Hyperlipidemia, n (%)	224 (40)	175 (37)	49 (55)	0.001^b^
COPD, n (%)	132 (24)	105 (22)	27 (30)	0.097^b^
High-risk surgery, n (%)	294 (52)	247 (52)	47 (53)	0.919^b^
Ischemic heart disease, n (%)	104 (19)	75 (16)	29 (33)	<0.001^b^
Congestive heart disease, n (%)	175 (31)	132 (28)	43 (48)	<0.001^b^
Cerebrovascular disease, n (%)	94 (17)	71 (15)	23 (26)	0.012^b^
Renal insufficiency, n (%)	40 (7)	33 (7)	7 (8)	0.765^b^
Insulin therapy for diabetes, n (%)	40 (7)	30 (6)	10 (11)	0.100^b^
Total RCRI, n (%)				0.001^b^
≤2	487 (87)	420 (89)	67 (75)	
>2	75 (13)	53 (11)	22 (25)	
Crystalloids, median (IQR)	2511 (2000-4206)	2500 (2000-4148)	3000 (2000-4950)	0.324^a^
Colloids, n (%)	176 (31)	143 (30)	33 (37)	0.201^b^
Erythrocytes, n (%)	172 (31)	138 (29)	34 (38)	0.090^b^
Fresh frozen plasma, n (%)	14 (3)	8 (2)	6 (7)	0.005^b^
Platelets, n (%)	3 (1)	2 (0.4)	1 (1)	0.405^b^
Outcomes:				
APACHE II score, median (IQR)	8 (5-11)	8 (5-10)	9 (7-12)	<0.001^a^
SICU length of stay (hours), median (IQR)	19 (16-30)	19 (15-23)	23 (18-71)	<0.001^a^
Hospital length of stay (days), median (IQR)	12 (5-24)	11 (5-23)	18 (9-35)	<0.001^a^
Mortality in SICU, n (%)	3 (1)	1 (0.2)	2 (2)	0.067^b^
Mortality in hospital, n (%)	30 (5)	15 (3)	15 (17)	<0.001^b^
Mortality at six-month follow-up, n (%)	74 (13)	46 (10)	28 (32)	<0.001^b^

^a^Mann-Whitney *U* test, ^b^Pearson *χ*^2^. IQR*,* interquartile range; ASA, American Society of Anesthesiologists; SICU, surgical intensive care unit; COPD, chronic obstructive pulmonary disease; RCRI*,* Revised Cardiac Risk Index; APACHE II*,* Acute Physiology and Chronic Health Evaluation II.

Six months after SICU discharge, 130 patients (27%), did not answer the questionnaires but were known to be alive. There were significant differences between respondents (n = 410) and nonrespondents (n = 162) with regard to the following background and SICU variables: dyslipidemia (43% vs. 30% respectively, *P* = 0.008), BMI (median, 25.4 vs. 24.4, *P* = 0.009), temperature at SICU admission (median, 34.9°C vs. 35.1°C, *P* = 0.041), type of anesthesia (locoregional anesthesia, 18% vs. 10%, *P* = 0.041), and administration of erythrocytes during surgery (27% versus 36%, *P* = 0.030).

Table 1 describes the preadmission variables in patients with and without postoperative delirium. The results of multivariate analysis for mortality at hospital and for mortality at six-month follow-up are provided in Tables 2 and 3. A logistic regression model identified delirium and age as variables significantly correlated with hospital mortality. Delirium, female gender, and congestive heart disease were significantly correlated with mortality at the six-month follow-up (Table 3).

**Table 2:** Odds ratios (OR) and respective 95% confidence intervals (CI) from multivariate analysis with logistic regression model

	**Dead in hospital**	
	OR adjusted*	**(95% CI)**
Delirium	2.673	(1.115; 6.407)
Age in years	1.071	(1.023; 1.121)
BMI	0.947	(0.860; 1.044)
ASA physical status III/IV	0.912	(0.306; 2.721)
COPD	1.432	(0.576; 3,561)
Troponin I at SICU admission	1.083	(0.667; 1.758)
Ischemic heart disease	0.875	(0.222; 3.444)
Congestive heart disease	1.301	(0.489; 3.459)
Total RCRI >2	1.438	(0.330; 6.273)
Fresh frozen plasma	3.540	(0.617; 20.20)
Platelets	5.613	(0.351; 89.6)
APACHE II score	1.025	(0.923; 1.138)

*Adjusted for all variables presented in the table. BMI, body mass index; ASA, American Society of Anesthesiologists; COPD, chronic obstructive pulmonary disease; SICU, surgical intensive care unit; RCRI, Revised Cardiac Risk Index; APACHE II, Acute Physiology and Chronic Health Evaluation II.

**Table 3:** Odds ratios (OR) and respective 95% confidence intervals (CI) from multivariate analysis with logistic regression model

	**Dead in six months**	
	OR adjusted*	**(95% CI)**
Delirium	2.562	(1.360; 4.828)
Female gender	1.897	(1.060; 3.395)
ASA physical status III/IV	0.731	(0.381; 1.402)
High-risk surgery	1.672	(0.863; 3.240)
Ischemic heart disease	1.025	(0.388; 2.708)
Congestive heart disease	2.093	(1.062; 4.123)
Total RCRI >2	0.972	(0.331; 2.850)
Erythrocytes,	0.958	(0.503; 1.825)
Fresh frozen plasma	3.140	(0.804; 12.26)
Platelets	11.360	(0.677; 190.5)
Age in years	1.027	
BMI	0.983	(0.928; 1.041)
Duration of anesthesia	1.002	(1.000; 1.004)
Troponin I at SICU admission	1.090	(0.644; 1.848)
Crystalloids	1.000	(1.000; 1.000)
APACHE II score	1.045	(0.969; 1.127)

*Adjusted for all variables presented in the table. ASA, American Society of Anesthesiologists; RCRI, Revised Cardiac Risk Index; BMI, body mass index; SICU, surgical intensive care unit; APACHE II, Acute Physiology and Chronic Health Evaluation II.

### Functional capacity and ADL

On admission to the SICU, 7% of the patients were dependent with regard to at least one I-ADL and 19% were dependent with regard to at least one P-ADL. The patients with delirium were more likely to be I-ADL-dependent (16% vs. 6%, *P* = 0.002) and P-ADL-dependent (39% vs. 16%, *P* <0.001) than those without delirium. On admission, Katz and Lawton scores indicated that the patients with delirium were more ADL-dependent than those without delirium (*P* <0.001and *P* <0.001 respectively). Six months after SICU discharge, 19% of the patients were dependent with regard to at least one I-ADL, and 40% with regard to at least one P-ADL. The patients with postoperative delirium exhibited more I-ADL dependency (39% vs. 16%, *P* <0.001) and P-ADL dependency (54% vs. 38%, *P* <0.001). At this time point Katz and Lawton scores indicated that the patients with postoperative delirium were more I-ADL-dependent, and more P-ADL-dependent than patients without postoperative delirium (*P* <0.001and *P* <0.001 respectively).

The results of multivariate analysis for becoming dependent for both instrumental and personal ADL at the six-month follow-up are presented in Tables 4 and 5. Preadmission variables identified by the logistic regression model as significantly correlated with becoming P-ADL-dependent were delirium, and erythrocyte transfusion (Table 4). The only preadmission variable identified by the model as significantly correlated with becoming I-ADL-dependent was COPD (Table 5).

**Table 4:** Odds ratios (OR) and respective 95% confidence intervals (CI) from multivariate analysis with logistic regression model

	**Become dependent**	
	**Personal ADL**	
	OR adjusted*	**(95% CI)**
Delirium	2.188	(1.075; 4.455)
ASA physical status III/IV	1.104	(0.566; 2.153)
Hypertension	1.148	(0.569; 2.317)
Hyperlipidemia	0.898	(0.478; 1.688)
COPD	1.220	(0.632; 2.345)
Ischemic heart disease	1.178	(0.475; 3.923)
Congestive heart disease	1.545	(0.794; 3.007)
Cerebrovascular disease	1.739	(0.833; 3.630)
Renal insufficiency	1.651	(0.633; 4.307)
Total RCRI >2	0.681	(0.229; 2.030)
Erythrocytes	1.906	(1.026; 3.541)
Fresh frozen plasma	2.712	(0.563; 13.06)
Age in years	1.009	(0.983; 1.036)
BMI	1.025	(0.947; 1.109)
APACHE II score	1.025	(0.972; 1.081)

*Adjusted for all variables presented in the table. ADL, activities of daily living; ASA, American Society of Anesthesiologists; COPD, chronic obstructive pulmonary disease; RCRI, Revised Cardiac Risk Index; BMI, body mass index; APACHE II, Acute Physiology and Chronic Health Evaluation II.

**Table 5:** Odds ratios (OR) and respective 95% confidence intervals (CI) from multivariate analysis with logistic regression model

	**Become dependent**	
	**Instrumental ADL**	
	OR adjusted*	**(95% CI)**
Delirium	0.786	(0.394; 1.570)
Type of anesthesia locoregional	1.414	(0.663; 3.015)
COPD	2.152	(1.266; 3.659)
Cerebrovascular disease	0.798	(0.373; 1.710)
Total RCRI >2	0.661	(0.316; 1.381)
Erythrocytes,	1.239	(0.732; 2.098)
Fresh frozen plasma	3.942	(0.722; 21.53)
BMI	1.032	(0.988; 1.077)
APACHE II score	1.050	(0.991; 1.113)

*Adjusted for all variables presented in the table. ADL, activities of daily living; COPD, chronic obstructive pulmonary disease; RCRI, Revised Cardiac Risk Index; BMI, body mass index, APACHE II, Acute Physiology and Chronic Health Evaluation II.

### Quality-of-life measures

The results of multivariate linear analysis for changes in quality of life in each SF-36 domain are provided in Table 6. The variable identified by the linear regression model as significantly correlated with changes in quality of life in the physical functioning domain was delirium. In the general health perception domain, changes were associated with total RCRI >2, and cerebrovascular disease. In the vitality domain, changes were associated with delirium, female gender, anesthesia (locoregional), cerebrovascular disease, and total RCRI >2. For social function, changes were associated with delirium, hypertension, and the use of fresh frozen plasma and platelets, and in the mental health domain, troponin I at SICU admission was associated with these changes. With regard to physical role function, bodily pain, and emotional role domains, none of the variables investigated correlated significantly with changes in quality of life. Included as independent variables in the models were the following variables: age in years, gender, ASA-PS, BMI, duration of anesthesia, type of anesthesia, locoregional anesthesia, emergency surgery, temperature at SICU admission, troponin I at SICU admission, hypertension, hyperlipidemia, COPD, high-risk surgery, ischemic heart disease, congestive heart disease, cerebrovascular disease, renal insufficiency, insulin therapy for diabetes, total RCRI, crystalloids, colloids, erythrocytes, fresh frozen plasma, platelets, and APACHE II scores. In all models, we found no indication of multicolinearity between variables; in fact, all VIF values were >3.

**Table 6:** Significant regression coefficients from linear regression* models with changes in SF-36 dimensions as dependent variables

**Changes in SF-36 dimension:**	**β**	***P***
Physical functioning:		
Delirium	17.402	0.007
General health perception:		
Total RCRI >2	17.702	0.030
Cerebrovascular disease	−11.348	0.049
Vitality:		
Delirium	8.221	0.015
Female gender	−5.488	0.005
Anesthesia locoregional	−7.910	0.007
Cerebrovascular disease	−9.389	0.015
Total RCRI >2	12.687	0.020
Social function:		
Delirium	16.805	0.005
Hypertension	14.610	0.004
Fresh frozen plasma	−29.931	0.028
Platelets	−81.884	0.024
Mental health:		
Troponin at SICU admission	44.593	0.005

*Adjusted for age, gender, American Society of Anesthesiologists (ASA) physical status, body mass index (BMI), duration of anesthesia, type of anesthesia, locoregional anesthesia, emergency surgery, temperature at surgical intensive care unit (SICU) admission, troponin I at SICU admission, hypertension, hyperlipidemia, chronic obstructive pulmonary disease (COPD), high-risk surgery, ischemic heart disease, congestive heart disease, cerebrovascular disease, renal insufficiency, insulin therapy for diabetes, total Revised Cardiac Risk Index (RCRI), crystalloids, colloids, erythrocytes, fresh frozen plasma, platelets, and Acute Physiology and Chronic Health Evaluation II (APACHE II) scores.

## Discussion

It is well known and well documented that the development of delirium after surgery is an important predictor of increased mortality; it has been reported that delirium is an independent risk factor for mortality even after controlling for pre-existing comorbidities, severity of illness, comatose state, and the use of sedative and analgesic medications [3,25-29].

The impact of postoperative delirium in outcome particularly in postdischarge mortality was confirmed in a recent meta-analysis by Witlox *et al.*[30] denoting the evidence that delirium is associated with long-term poor outcome.

In the recently published *Clinical practice guidelines for the management of pain, agitation, and delirium in adult patients in the intensive care unit*, Barr *et al.*[7] put an emphasis on the relation between delirium and mortality reporting that several prospective cohort studies examined the relationship between delirium while in the ICU and mortality at various time points after ICU discharge, concluding that delirium was an independent predictor of mortality in several studies.

In agreement with these reports, the current data report that a large number of patients with postoperative delirium died before and after discharge from the ICU, and delirium was considered an independent risk factor for mortality at the hospital, and at six months after discharge from the SICU. Indeed, the patients with postoperative delirium had higher mortality rates: 10 times higher at SICU, almost 6 times higher at hospital discharge, and 3.2 times higher at the six-month follow-up.

Others have found that risk factors for delirium are associated with poor outcome [31-33]. In this study, postoperative delirium was associated with mortality, and was considered to be an independent predictor of mortality at hospital discharge and at six-month follow-up. Beyond delirium, we identified age as an independent predictor of hospital mortality and female gender and congestive heart disease as independent predictors of mortality at six-month follow-up. Hutt *et al.*[34] reported a higher prevalence of delirium in patients with heart failure (35.3% of 156 nursing-home residents), and that there was an association with a threefold increased risk of 60-day mortality. Uthamalingam *et al.*[35], in their study in congestive heart disease patients, showed that delirium was independently associated with increased 30- and 90-day readmission, and short-term mortality, after adjusting for potential confounders.

Delirium may be viewed as an indicator of poor outcome after surgery. Our results indicated that postoperative delirium was an independent risk factor for becoming dependent in P-ADL six months after discharge from the SICU. These results are in agreement with those reported by Olofsson *et al.*[36], who concluded that patients with delirium were more dependent with regard to ADL upon discharge, and four months after discharge. Schuurmans *et al.*[37] and Marcantonio *et al.*[38] have suggested that surgical patients with hip fractures who exhibited more ADL dependency before the fracture were at greater risk of developing delirium. In our study, postoperative delirium was a risk factor for becoming dependent in P-ADL, but we did not observe the same results for I-ADL. As P-ADL are related to self-care tasks, P-ADL dependency represents a profound impact on health.

The results of our study suggested that postoperative delirium has an important impact on the quality of life. In addition, the patients who experienced delirium while in hospital were prone to worsening of some domains of quality of life, as compared to those without postoperative delirium. Our results are in accordance with the hypothesis proposed by Inouye [39], that there is individual vulnerability in each patient, which, in combination with a precipitating factor (surgery), may result in the development of delirium. Our study suggested that postoperative delirium is an independent predictor of poor results in three of the eight SF-36 domains, using a methodology that evaluates overall change for each SF-36 domain. The domains of the SF-36 in which delirium predicted a worsening of results after six months were physical function, vitality, and social function, domains that involve both mental and physical aspects of quality of life.

Although delirium is increasingly being recognized as a common, serious, and potentially preventable cause of morbidity and mortality [40], it has received little attention and continues to be underestimated with respect to the influence it has on quality of life. Duppils *et al.*[8] concluded that patients who experienced delirium after a hip fracture scored lower at follow-up in the physical functioning and vitality subscales of the SF-36 than those who did not experience delirium. However, their study consisted of a very small population of elderly orthopedic patients, and the results were not adjusted for other covariates, as they were in our present study. In an intensive care patient population, Van Rompaey *et al.*[6] reported that patients with delirium exhibited lower results than patients without delirium at the six-month follow-up; however, their study was only a comparative analysis and no adjustments were incorporated into the analysis.

We did not record sedative doses administered during the ICU stay, and these may have influenced the occurrence of delirium. The lack of data regarding comorbidities occurring after discharge and the cognitive status of the patients six months after SICU discharge is also a limitation of the study; patients who experienced postoperative delirium may have been less likely to complete the six-month follow-up assessment. Supporting this remark are reports suggesting that there may exist an association between the presence of delirium in ICU and a higher incidence of cognitive dysfunction after ICU discharge [10,41] and we may hypothesize that this could have been one reason to not complete the follow-up.

Although some statistically significant differences were found between responders and nonresponders regarding some preoperative and surgery-related characteristics of the patients, this may reflect only a small bias, and does not invalidate the overall conclusions of the study.

## Conclusions

Postoperative delirium was an independent risk factor for both hospital mortality and mortality at six-month follow-up. It was also an independent risk factor for dependency in P-ADL after SICU discharge, and was significantly associated with a reduction in the quality of life.

## Key messages

•Patients with postoperative delirium had a higher mortality rate and postoperative delirium was an independent risk factor for mortality.

•Postoperative delirium was a risk factor for becoming dependent in P-ADL.

•Postoperative delirium was a determinant of worse quality of life six months after SICU discharge in three of the eight SF-36 domains: physical function, vitality, and social function.

## Abbreviations

ADL: Activities of daily living; APACHE: Acute Physiology and Chronic Health Evaluation; ASA-PS: American Society of Anesthesiologists Physical Status; BMI: Body mass index; CAM-ICU: Confusion Assessment Method for the intensive care unit; CI: Confidence interval; DSM: Diagnostic and Statistical Manual of Mental Disorders; HRQL: health-related quality of life; I-ADL: Instrumental activities of daily living; ICDSC: Intensive Care Delirium Screening Checklist; ICU: Intensive care unit; LOS: Length of stay; OR: Odds ratio; PACU: Postanesthesia care unit; P-ADL: Personal activities of daily living; RASS: Richmond Agitation and Sedation Scale; RCRI: Revised Cardiac Risk Index; SAPS: Simplified Acute Physiology Score; SD: Standard deviation; SF-36: Medical outcomes study short-form health survey; SICU: Surgical intensive care unit; VIF: Variance inflation factor.

## Competing interests

The authors did not use funds for the research and have no conflicts of interest.

## Authors’ contributions

All people listed as authors contributed to the preparation of the manuscript and no person or persons other than the authors listed have contributed significantly to its preparation. Each listed author participated in the work to the extent that they could all publicly defend its content. They all read the manuscript before its submission for publication and are prepared to sign a statement stating they had read the manuscript and agree to its publication. CL carried out the collection of data, preparation of the manuscript and writing of the manuscript; DV carried out the collection of data and preparation of the manuscript; DP carried out the collection of data and preparation of the manuscript; CS performed the statistical analysis and carried out the collection of data and preparation of the manuscript; VF carried out the collection of data and preparation of the manuscript; PS carried out the collection of data and preparation of the manuscript; MB carried out the collection of data and preparation of the manuscript; AS carried out the collection of data and preparation of the manuscript and FA conceived the study and carried out the preparation of the manuscript, analysis of data and writing of the manuscript.
